# 2235 15N-Leucine transport across the blood brain barrier is significantly impaired in the glutamine synthetase-inhibited brain

**DOI:** 10.1017/cts.2018.37

**Published:** 2018-11-21

**Authors:** Shaun E. Gruenbaum, Roni Dhaher, Kevin Behar, Hitten Zaveri, Mark Erfe, Tore Eid

**Affiliations:** Yale School of Medicine

## Abstract

OBJECTIVES/SPECIFIC AIMS: Astroglial glutamine synthetase (GS), which metabolizes glutamate and ammonia to glutamine, is critical for the detoxification of brain ammonia, clearance of synaptic glutamate, and production of brain glutamine. Perturbations in the expression and activity of GS are thought to play a causative role in the pathogenesis of several conditions of abnormal neurotransmission. Although the long-term consequences of GS inhibition on amino acid homeostasis in the brain are unknown, it is thought that amino acid influx in the brain is tightly coupled with glutamine efflux via the L-type amino acid transporter. Both glutamine and leucine serve many critical functions in the brain including protein synthesis, gene expression, insulin regulation, and immune signaling. The objective of this study was to determine the effects of chronic GS inhibition with methionine sulfoximine (MSO) on glutamine and leucine homeostasis in the brain. METHODS/STUDY POPULATION: In total, 12 rats were surgically implanted with microdialysis guide cannulas in the bilateral dentate gyrus. Rats were randomly divided for surgical implantation of either a MSO (n=6) or phosphate buffer saline (PBS; n=6) pump in the right dentate gyrus. After 7 days, bilateral microdialysis probes were placed under brief isoflurane anesthesia, and microdialysis flow was established by infusing 0.5 µL/min of artificial extracellular fluid. Dialysate samples were collected every 30 minutes for the duration of the experiment. A 113 mM 15N-Leucine (3.6 mL/h) and 2 M 2–13C-sodium acetate (0.0633 μL/g/min for *t*=0–5 min, 0.0316 μL/g/min for *t*=5–10 min, and 0.0253 μL/g/min for *t*>10 min) solution was infused intravenously for 300 minutes. The EZ:Faast Free Amino Acid analysis kit and ultra-performance liquid chromatography/tandem mass spectrometry was used for quantification of amino acids in the dialysate fluid. RESULTS/ANTICIPATED RESULTS: At baseline (*t*=0 h), the concentrations of glutamine were significantly lower in MSO-treated rats (*p*<0.001) in the ipsilateral (GS-inhibited) hippocampus. There were no differences in glutamine concentrations between MSO and PBS-treated rats in the contralateral hippocampus. In PBS-treated rats, there was a significant increase in 15N-leucine between *t*=0 hour and *t*=5 hour in the contralateral (*p*<0.05) and ipsilateral (*p*<0.05) hippocampus. In MSO-treated rats, there was a significant increase in 15N-leucine between *t*=0 and *t*=5 hours in the contralateral (*p*<0.05) hippocampus, but not in the ipsilateral hippocampus (*p*=ns.). DISCUSSION/SIGNIFICANCE OF IMPACT: This study demonstrated for the first time that basal glutamine concentrations are low in areas of the brain where GS is acutely inhibited, and that leucine uptake in these brain areas are markedly decreased. Perturbations in glutamine and leucine homeostasis have been implicated in several disease processes including diabetes, obesity, liver disease, immune system dysfunction, epilepsy, and cancer, and the glutamine-dependent leucine influx in the brain may be a novel and important therapeutic target to treat these conditions.

